# Predicting Intense Levels of Child Anxiety During Anesthesia Induction at Hospital Arrival

**DOI:** 10.1007/s10880-020-09716-6

**Published:** 2020-04-18

**Authors:** Robin Eijlers, Lonneke M. Staals, Jeroen S. Legerstee, Johan M. Berghmans, Elske M. Strabbing, Marc P. van der Schroeff, René M. H. Wijnen, Laura S. Kind, Manon H. J. Hillegers, Bram Dierckx, Elisabeth M. W. J. Utens

**Affiliations:** 1 Department of Child and Adolescent Psychiatry/Psychology, Erasmus Medical Centre, Sophia Children’s Hospital, Wytemaweg 8, Rotterdam, 3015 CN The Netherlands; 2 Department of Anaesthesiology, Erasmus Medical Centre, Sophia Children’s Hospital, Rotterdam, The Netherlands; 3 Department of Anaesthesia, ZNA Middelheim, Queen Paola Children’s Hospital, Antwerp, Belgium; 4 Department of Oral and Maxillofacial Surgery, Erasmus Medical Centre, Sophia Children’s Hospital, Rotterdam, The Netherlands; 5 Dutch Craniofacial Centre, Erasmus Medical Centre, Sophia Children’s Hospital, Rotterdam, The Netherlands; 6 Department of Otorhinolaryngology and Head and Neck Surgery, Erasmus Medical Centre-Sophia Children’s Hospital, Rotterdam, The Netherlands; 7 Intensive Care and Department of Paediatric Surgery, Erasmus Medical Centre-Sophia Children’s Hospital, Rotterdam, The Netherlands; 8 Centre for Special Care Dentistry, CBT Rijnmond, Rotterdam, The Netherlands; 9 Research Institute of Child Development and Education, University of Amsterdam, Amsterdam, The Netherlands; 10 Academic Centre for Child Psychiatry De Bascule, Department of Child and Adolescent Psychiatry, Academic Medical Centre, Amsterdam, The Netherlands

**Keywords:** General anesthesia, Anxiety, Child, Psychometrics, Diagnostic techniques and procedures

## Abstract

In children, intense levels of anxiety during anesthetic induction are associated with a higher risk of pain, poor recovery, and emergence delirium. Therefore, it is important to identify these high-risk children at hospital arrival. The current study examined internalizing behavior (Child Behavior Checklist, CBCL) and state anxiety measures (modified Yale Preoperative Anxiety Scale, mYPAS, and State Trait Anxiety Inventory for Children, STAIC) at hospital arrival as predictors of anxiety during induction of anesthesia. One hundred children (aged 4 to 12 years) undergoing elective daycare surgery were included. The STAIC and mYPAS at hospital arrival were significant predictors of anxiety during induction, whereas CBCL was not. The STAIC state form at hospital arrival was the strongest predictor and could be used to identify children who will experience intense levels of anxiety during anesthetic induction, with sufficient to good diagnostic accuracy. Using the STAIC at hospital arrival allows targeted interventions to reduce anxiety in children.

## Introduction

To some extent, most children experience anxiety on the day of surgery, but the range of anxiety intensity is wide (Kain et al. 1996). Preoperative anxiety peaks during induction of anesthesia and is associated with different maladaptive consequences after surgery. For example, in a study by Kain et al. (2006) including 241 5- to 12-year-old children undergoing elective surgery, anxious patients had significantly higher levels of pain in the recovery room, as well as during the first 3 days after surgery. As a result, anxious children also needed more analgesia. These findings have been replicated by numerous studies (Chorney and Kain 2009; Kain et al. 2007; Power et al. 2012). Preoperative anxiety is also related to the occurrence of emergence delirium in the recovery room (Berghmans et al. 2015; Dahmani et al. 2014; Kain et al. 2006; Kain et al. 2004a, b; Malarbi et al. 2011). Children experiencing emergence delirium can be extremely agitated, hypersensitive to stimuli, do not recognize surroundings or people, and are inconsolable when emerging from general anesthesia (Aldecoa et al. 2017). Previous studies have also found that preoperative anxiety is associated with psychological problems and negative behavioral changes in the 2 weeks after surgery, including apathy, anxiety, sleeping disturbances, and aggression toward authority (Kain et al. 2006, 1996; Maclaren and Kain 2007). Children experiencing intense levels of preoperative anxiety (defined as one SD above the mean) are particularly at risk for these maladaptive consequences (Kain et al. 2004a, b). In addition, Ben-Amitay et al. (2006) found that preoperative anxiety in 6- to 18-year-old patients (*n* = 40) was associated with posttraumatic stress symptoms after elective surgery, which persisted at the follow-up assessments, 1 month and 6 months after surgery, in respectively 8% and 5% of all patients. Finally, childhood healthcare experiences have found to be predictive of adolescents’ healthcare behavior (e.g., attending checkups). Therefore, negative hospital experiences, including perioperative anxiety and low comfort, could result in low adherence to future medical treatment (Byrne 2008; Forrest et al. 2004; Jones et al. 2008). These experiences can even impact adult healthcare use, as it has been found that adults with negative memories about childhood hospital experiences are more likely to avoid healthcare as an adult (Pate et al. 1996).

Considering these adverse consequences of elevated preoperative anxiety levels, it is important to enable clinicians to distinguish high-risk children from other children. Different strategies have been proposed to reduce anxiety during induction of anesthesia (Manyande et al. 2015). While some non-pharmacological interventions, such as information materials (Smith and Callery 2005) can be easily provided to all children; other interventions, such as comprehensive coping programs (Watson et al. 2002), come with increased costs in terms of time and money. Hence, targeted intervention is desirable, but this first requires the identification of high-risk patients.

Fortier et al. (2011) found that internalizing behavior in the past 6 months (e.g. anxiety or depression), as indicated by the widely used Child Behavior Checklist (CBCL), could predict anxiety during induction of anesthesia in adolescents (*n* = 59). Recently, Berghmans et al. (2015) found that internalizing behavior also predicted anxiety during induction in children (*n* = 401). The relationship between internalizing behavior and anxiety during induction of anesthesia is a fairly unexplored research area, so it is important to further expand this promising line of research.

State anxiety at hospital arrival has a much closer temporal association with children’s anxiety during anesthetic induction than internalizing behavior in the past 6 months. Acquiring information on state anxiety directly at hospital arrival (prior to admission) allows parents or healthcare professionals to intervene before anxiety possibly increases. The gold standard for observational assessment of preoperative anxiety is the modified Yale Preoperative Anxiety Scale (mYPAS) (Kain et al. 1997). Another well-validated tool to assess state anxiety is the State Trait Anxiety Inventory for Children (STAIC) (Spielberger and Edwards, 1973). This self-report tool is often very difficult to use for young children (≤ 8-year old), as it is considered too lengthy and complex (Schisler et al. 1998). Parents can complete the questionnaire about their children’s state anxiety as an alternative (Creswell et al. 2008).

Identifying high-risk children, well before entering the operating room, may improve induction of anesthesia. Therefore, the aim of the current study was to examine standardized tools (the CBCL, mYPAS, and STAIC) at hospital arrival to identify a high-risk population, who would experience intense levels of anxiety during induction (mean mYPAS + 1 SD). We hypothesized that levels of internalizing behavior, as measured with the CBCL, as well as state anxiety, as measured with the mYPAS and STAIC, at hospital arrival are predictors of anxiety during induction of anesthesia. Furthermore, we expected that state anxiety at hospital arrival would be a stronger predictor of anxiety during induction of anesthesia, because state anxiety at hospital arrival has a closer temporal association with anxiety during induction of anesthesia than internalizing behavior in the past 6 months.

## Methods

This study is part of a single blinded randomized controlled trial to investigate the efficacy of a preoperative virtual reality intervention on anxiety, pain, and emergence delirium (Eijlers et al.^2017^) (Netherlands Trial Registry: NTR6116, https://www.trialregister.nl/trial/5935). For the purpose of this paper, only children who received care as usual (CAU) were included. Preoperative preparation of the intervention group differed considerably from regular care and was thus not included in the current study. The study was conducted at the Erasmus Medical Center, Sophia Children’s Hospital in Rotterdam, The Netherlands, and has been approved by the Medical Ethics Committee of the Erasmus Medical Center (MEC-2016–626). Eligible participants were consecutive pediatric patients (aged 4 to 12 years) undergoing elective daycare surgery (i.e. maxillofacial, dental, or ear-nose-throat surgery) between March 2017 and October 2018. Exclusion criteria were intellectual disabilities (i.e. IQ < 70), inability of parents to read or write Dutch, epilepsy, visual impairment, poor general health, indicated by the American Society of Anesthesiologists (ASA) physical status ≥ III, or preoperative anxiolytic medication.

### Procedure

During the preoperative screening visit, pediatric anesthesiologists informed eligible patients and parents about the study. If they were prepared to cooperate with the study, they received the patient information folder via email. During this preoperative screening visit, anesthesiologists provided comprehensive educational information concerning general anesthesia and recommended that all children and parents should watch an informative online movie at home about general anesthesia, as per the standard hospital protocol. On the day of surgery, at hospital arrival, written informed consent was obtained from all parents, as well as from all children aged 12 years. Children aged 11 years and under provided oral consent for participation in the study.

At hospital arrival (T1), in the main entrance hall, parents completed the CBCL (child’s internalizing behavior), STAIC (child’s state anxiety), and STAI (own state anxiety). In addition, the research assistant administered the mYPAS and collected demographical/medical data (T1). Parental education (i.e. highest level of education of either parent) was used as an indicator of socioeconomic status (SES) and was categorized into low (= 1), middle (= 2) or high (= 3), according to Statistics Netherlands (Centraal Bureau voor de Statistiek: https://statline.cbs.nl). Once these data had been collected, the children were admitted to the daycare unit.

After admission, children and one accompanying parent were brought to the preoperative holding area, approximately one and a half hours after hospital arrival. From here, an anesthesiologist and anesthetic nurse transported the child and parent to the operating room. In the operating room, a researcher who was blinded to T1 assessments evaluated anxiety during induction of anesthesia (mYPAS T2). Both the research assistant and researcher were trained in administering the mYPAS with standardized instructions. Interrater reliability was excellent (Cronbach’s alpha 0.90, based on 30 double observations).

### Anesthesia Protocol

Preoperatively, none of the children received anxiolytic premedication. Cream with local anesthetics, e.g. EMLA, was applied on the back of the hands, 30–60 min before transportation to the operating room. Anesthetic preparation (i.e. placement of electrocardiography electrodes, pulse oximeter, and blood pressure cuff) took place in the operating room. Anesthetic induction was performed intravenously (IV). If IV placement was not preferred or IV access was not successful, anesthesia was achieved by inhalation induction. For IV induction, a peripheral IV catheter was placed on the back of the hand, and propofol (2–4 mg ⋅ kg^−1^ IV) and fentanyl (1–2 mcg kg^−1^ IV) were administered. For inhalation induction, sevoflurane in a mixture of oxygen and air was administered by mask. In these cases, IV placement took place after induction, after which fentanyl (1–2 mg kg^−1^ IV) was administered.

### Assessment Instruments

#### Modified Yale Preoperative Anxiety Scale

The mYPAS is considered to be the gold standard to assess preoperative anxiety (in the holding area and operating room) (Kain et al.^1997^). This observational instrument consists of 27 items covering five domains: activity, emotional expressivity, state of arousal, vocalization, and use of parents. Scores range from 23.33 to 100.00, with higher scores indicating higher levels of anxiety. The domains have good to excellent inter-observer and intra-observer reliability and good validity (Chorney and Kain, 2009; Kain et al.^1997^). The mYPAS was administered both at hospital arrival (T1) and during anesthetic induction (T2).

#### Child Behavior Checklist

The CBCL, concerning emotional and behavioral problems in the past 6 months, was used to assess internalizing behavior, i.e. anxious/depressed, withdrawn-depressed, and somatic complaints in children (Achenbach and Rescorla 2000, 2001). Either the 1^1/2^–5 years of age version with 100 items (for 4- to 5-year-old participants) or the 6–18 years of age version with 113 items (for 6- to 12-year-old participants) was completed by parents at hospital arrival. Response categories range from 0 to 2. Summary scores were computed, with higher scores indicating more problems. As the internalizing scale consists of 36 items for the 1^1/2^ years of age version and of 32 items for the 6–18 years of age version, the summary scores of 6- to 12-year-old participants were multiplied by 1.125 (36/32) to obtain a total score of the CBCL for different ages. The widely used CBCL has good to excellent validity and reliability (Achenbach et al.^2008^).

#### State Trait Anxiety Inventory for Children

The state anxiety form of the STAIC questionnaire was used to assess situational anxiety at hospital arrival (Spielberger and Edwards 1973). Twenty questions were answered on a 4-point Likert scale. Scores range from 20 to 80, with higher scores indicating higher levels of anxiety. As the sample included young children, a parent-reported version of the questionnaire was used (Creswell et al. 2008). The internal consistency of the STAIC in the current sample was excellent (Cronbach’s alpha = 0.91).

#### State Trait Anxiety Inventory

Parents completed the state anxiety form of the STAI questionnaire to assess their own situational anxiety at hospital arrival. The STAI consists of 20 questions with a 4-point Likert scale and has good validity and reliability (Spielberger and Gorsuch, 1983).

### Statistical Analysis

Baseline characteristics and psychological assessments were reported as mean (SD) or, in case of categorical data, as frequency (percentage). To test for multicollinearity between predictor variables (Pearson’s *r* ≥ 0.8), a correlation matrix and variance inflation (VIF) factors were computed. Univariate analyses were performed to test for significant associations between predictor and outcome variables.

The aim was to examine whether internalizing behavior and state anxiety at hospital arrival (T1) could predict intense levels of anxiety during induction of anesthesia (T2). First, a hierarchical multiple regression analysis was conducted with mYPAS at T2 as a dependent variable and CBCL summary scores for internalizing behavior, STAIC, and mYPAS at T1 as the predictors. The model was adjusted for age, sex, and SES. Second, for the strongest predictor in this model, receiver operating characteristic (ROC) curve analyses were performed to determine a cut-off value for high-risk patients, who will experience intense anxiety during induction of anesthesia (mean mYPAS T2 + 1 SD). The optimal cut-off point was found using Youden index *J* (sensitivity + specificity − 1). All data were analyzed with SPSS 24.0 for Windows. A *p *value < 0.05 was considered statistically significant.

### Sample Size Calculation

A sample size of 100 patients was sufficient to perform multiple linear regression analyses using 6 predictors at hospital arrival (T1) to predict anxiety during induction of anesthesia (mYPAS T2), with a power of 0.85 and an alpha of 0.05 to detect a small to medium effect size.

## Results

### Patient Characteristics

Between March 2017 and October 2018, 393 children were assessed for eligibility for the overall study. In total, 193 children did not participate, because they did not meet the inclusion criteria (*n* = 35), did not want to participate (*n* = 109), or for other reasons, e.g. unable to get in contact with, or their surgery was postponed (*n* = 49). Two hundred children were enrolled (VR intervention: *n* = 100, CAU: *n* = 100) (Eijlers et al.2019a, b) The current study only comprised children in the CAU condition. Two children were excluded either because of non-compliance with the anesthetic protocol (*n* = 1) or because no data were collected during anesthetic induction, due to logistical reasons (*n* = 1). Consequently, 98 participants were included in the data analysis.

Patient characteristics and assessment outcomes are given in Table 1. The mean age of all participants was 8.0 years and 58.2% of the participants were male. The majority of the participants (67.3%) had a physical status of ASA I, whereas 32.7% had a physical status of ASA II. Most participants (66.3%) had never had surgery before. Inhalation and intravenous induction were performed equally often (52.0% and 48.0%, respectively). Most children had parents with high SES (61.2%). No gender differences were found in these patient characteristics.

**Table 1:** Patient characteristics and baseline variables

	Children (*n* = 98)
Age	8.0 (2.8)
Sex	
Male	57 (58.2)
Female	41 (41.8)
ASA physical status	
I	66 (67.3)
II	32 (32.7)
Type of surgery	
Adenoidectomy and/or tonsillectomy	23 (23.5)
Tympanostomy tubes	23 (23.5)
Maxillofacial and dental procedures	26 (26.5)
Other ENT procedures	26 (26.5)
Previous surgery (yes)	33 (33.7)
Induction method	
Inhalation	51 (52.0)
Intravenously	47 (48.0)
SES	
Low	2 (2.0)
Medium	36 (36.7)
High	60 (61.2)
STAI parent –(T1)	36.1 (9.5)
CBCL internalizing behavior (T1)	6.0 (6.8)
STAIC (T1)	41.3 (9.3)
mYPAS (T1)	29.3 (8.2)
mYPAS (T2)	45.4 (22.4)

Values are mean (SD) or frequency (percentage). *ASA* American Society of Anesthesiologists; *ENT* ear, nose, throat; *SES* socioeconomic status; *STAI* state-trait anxiety inventory; *CBCL* child behavior checklist; *STAIC* state-trait anxiety inventory for children; *mYPAS* modified Yale preoperative anxiety scale. T1: at hospital arrival. T2: during induction of anesthesia

### Univariate Analysis

Univariate results are given in Table 2. Pearson’s correlation demonstrated that higher levels of state anxiety at hospital arrival (both STAIC T1 and mYPAS T1) were significantly associated with higher levels of anxiety during anesthetic induction (mYPAS T2) (*r* = 0.32 and *r* = 0.33, respectively). No association was found between internalizing behavior (CBCL T1) and anxiety during anesthetic induction (mYPAS T2) (*r* = 0.11).

**Table 2:** Univariate associations (Pearson’s *r*) between predictors and anxiety during induction of anesthesia

Predictors	Outcome mYPAS T2
Age	− 0.09
Sex	0.03
SES	0.00
CBCL internalizing behavior T1	0.11
STAIC T1	0.32**
mYPAS T1	0.22*

*SES* socioeconomic status; *CBCL* child behavior checklist; *STAIC* state-trait anxiety inventory for children; *mYPAS* modified Yale preoperative anxiety scale. T1: at hospital arrival. T2: during induction of anesthesia

^*^p < 0.05, **p < 0.01

### Multivariate Analysis

VIF factors were low (maximum 1.23) and no multicollinearity was found between predictor variables. Parent-reported state anxiety (STAIC, *p* = 0.001) and observer-reported state anxiety (mYPAS, *p* = 0.048) at hospital arrival (T1) were significant independent predictors of anxiety during anesthetic induction (mYPAS T2), when correcting for all other variables (*R*^2^ = 0.17, *F* = 3.20, *p* = 0.007) (Table 3). Internalizing behavior (CBCL T1) was not a significant predictor of anxiety during induction (*p* = 0.964). The strongest predictor, associated with the largest standardized beta coefficient, was STAIC (*β* = 0.35).

**Table 3:** Multiple regression: predictors of anxiety during induction of anesthesia

	Outcome: mYPAS T2							
Predictors	Model 1		Model 2		Model 3		Model 4	
	*B*	*β*	*B*	*β*	*B*	*β*	*B*	*β*
Constant	52.070**		47.786**		11.436		-0.586	
Age	− .748	− .093	− .795	− .098	− 1.408	− .174	− 1.611*	− .199
Sex	1,044	.023	1.003	.022	− 1.423	− .031	.141	.003
SES	− .428	− .010	.467	.011	3.042	.073	2.969	.071
CBCL internalizing behavior T1			.394	.119	.120	.036	.015	.005
STAIC T1					.903**	.373	.847**	.350
mYPAS T1							.550*	.201
*R*^2^	0.009		0.023		0.138		0.174	
*R*^2^ change	0.009		0.014		0.115		0.036	
*F*	0.292 (*p* = 0.831)		0.544 (*p* = 0.704)		2.944* (*p* = 0.016)		3.202** (*p* = 0.007)	

*SES* socioeconomic status; *CBCL* child behavior checklist; *STAIC* state-trait anxiety inventory for children; *mYPAS* modified Yale preoperative anxiety scale. T1: at hospital arrival. T2: during induction of anesthesia

*n* = 98, *p < 0.05, **p < 0.01

### Cut-off on Anxiety at Hospital Arrival

**Fig. 1:**
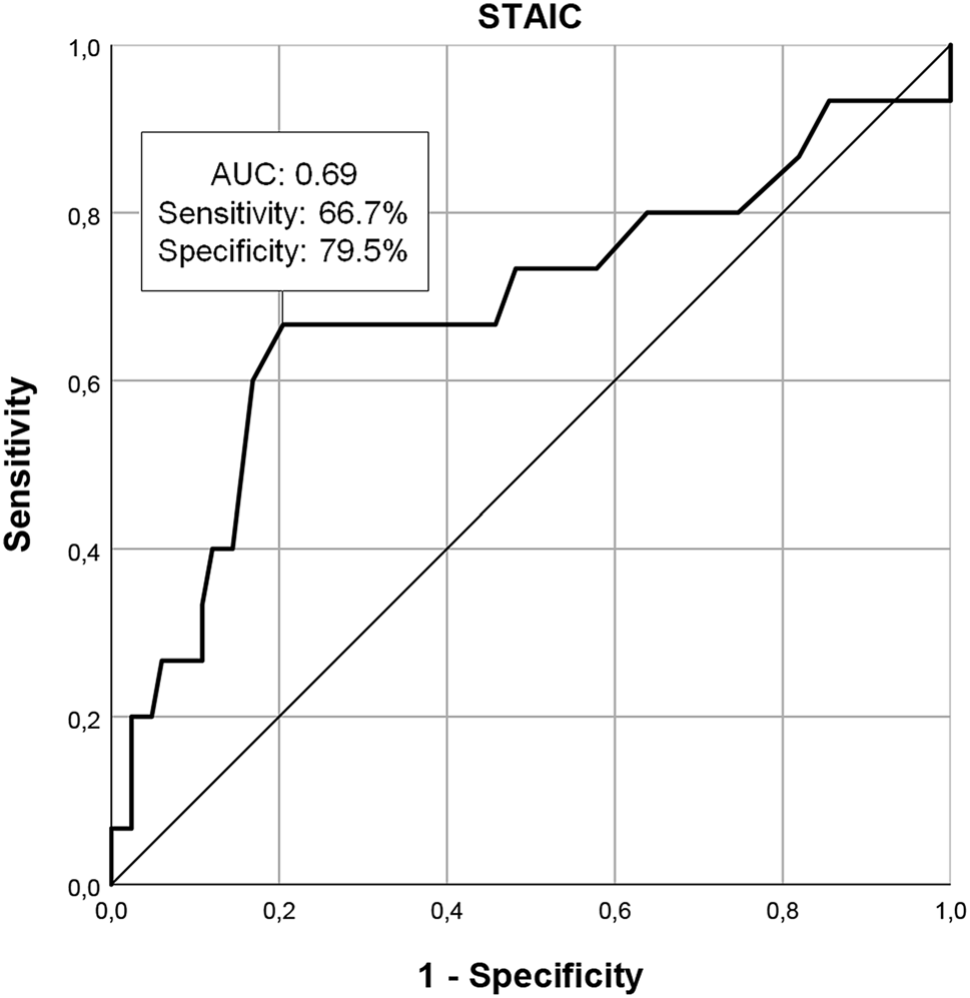
Receiver operating characteristic curve for State-Trait Anxiety Inventory for Children (STAIC) at hospital arrival (T1) for sensitivity and 1—specificity, including cut-off on the STAIC to distinguish between children who will experience intense levels of anxiety during induction of anesthesia (mYPAS, modified Yale Preoperative Anxiety Scale (mYPAS) T2 ≥ 67.79) and children who will not (mYPAS T2 < 67.79)

As the STAIC was the strongest predictor of anxiety during anesthetic induction, an optimal cut-off value on the STAIC was identified to predict intense levels of anxiety during induction (mean mYPAS T2 + 1 SD = 45.39 + 22.40 = 67.79). A total of 15 children (15.3%) experienced intense levels of anxiety during induction (mYPAS T2 ≥ 67.79). The ROC curve analysis identified a STAIC score of 47.50 as the optimal cut-off value to distinguish these high-risk patients from other patients (area under the curve (AUC) = 0.69, 95% CI = 0.52–0.86, *p* = 0.021) (Fig. 1). An AUC of 0.69 indicates sufficient to good diagnostic accuracy (Šimundić, 2009). For this cut-off value, sensitivity was 66.7% and specificity was 79.5%.

### Exploratory Analysis

No gender differences were found for internalizing behavior (*F* = 0.539, *p* = 0.465) or state anxiety, neither at hospital arrival (*F* = 2.208, *p* = 0.141 for STAIC and *F* = 2.554, *p* = 0.113 for mYPAS) nor during induction of anesthesia (*F* = 0.086, *p* = 0.770). Anxiety levels during induction of anesthesia were equal among different types of surgery (*F* = 0.231, *p* = 0.875). We have repeated the multivariate analysis in which we additionally corrected for parental state anxiety at T1 (STAI) and whether patients had previously undergone surgery. These results did not differ from the results of the original analysis (adjusted model 4: *R*^2^ = 0.180, *F* = 2.438, *p* = 0.020).

## Discussion

The aim of the current study was to examine standardized tools (the CBCL, mYPAS, and STAIC) at hospital arrival to identify a high-risk population, who would experience intense levels of anxiety during induction of anesthesia. The study was based on a sample of 98 children undergoing elective daycare surgery. Contrary to our hypotheses, internalizing behavior (CBCL) was not a significant predictor of anxiety during induction. However, both parent- and observer-reported state anxiety at hospital arrival (STAIC and mYPAS, respectively) were, as hypothesized, significant predictors of anxiety during induction. The STAIC (state form) was the strongest predictor and could be used to predict intense levels of anxiety (mean mYPAS during anesthesia induction + 1 SD), with sufficient to good diagnostic accuracy.

It is important for clinical practice to be able to predict levels of child anxiety during induction of anesthesia, because of the maladaptive postoperative consequences of preoperative anxiety, including increased pain, need for analgesia, and risk of emergence delirium (Chorney and Kain 2009; Dahmani et al. 2014; Kain et al. 2006, 2007, 1996; Malarbi et al. 2011; Power et al. 2012). Furthermore, highly anxious children are at risk of developing psychological problems in the 2 weeks following surgery, including anxiety, apathy, and sleeping disturbances, as well as posttraumatic stress symptoms that may persist up to 6 months (Ben-Amitay et al. 2006; Kain et al. 2006, 1996; Maclaren and Kain 2007). Finally, these negative experiences may lead to lower adherence to medical treatment and avoidance of future necessary healthcare (Byrne 2008; Forrest et al. 2004; Jones et al. 2008). These short-term and long-term maladaptive consequences of high levels of preoperative anxiety underscore the importance of identifying high-risk patients. Once these high-risk children have been identified, targeted psychological interventions can be provided. This is important, as it is often not feasible to apply anxiety-reducing interventions to all individual children undergoing surgery, due to the demands on time of clinical staff.

We were not able to replicate previous findings concerning the predictive value of the CBCL for anxiety during induction of anesthesia (Berghmans et al. 2015; Fortier et al. 2011). It is possible that differences in the distribution of SES played a role in this discrepancy between the findings, since it has been found that lower SES was related to higher CBCL scores (Van Oort et al. 2011). In the current study, parents with high SES were over-represented (61.2%). Age may be another explanatory factor. We focused on a more narrow age range (4 to 12 years) than Berghmans et al. (2015) (1.5 to 16 years) and a younger age range than Fortier et al. (2011) (11 to 18 years). The nature of preoperative anxiety strongly differs with age. Whereas younger children may have separation anxiety and fear of strangers, older children may fear pain after surgery and may be more aware of potential risks of undergoing surgery (Kain et al. 2000; Lumley et al. 1993). Therefore, the inconsistent results concerning the predictive value of CBCL on anxiety during induction might indicate a more complex relationship between age, anxiety during induction, and internalizing behavior. These contradictory results indicate the need for replication studies on this relationship.

Another possible explanation for our finding that internalizing behavior, as measured with the CBCL, was not predictive of levels of anxiety during induction of anesthesia is that the CBCL is a relatively broad-band measure, covering a variety of emotional and behavioral problems. In other words, the CBCL may not be sufficiently sensitive to detect those children who will be highly anxious during induction of anesthesia. Therefore, it would be useful to investigate if narrow-band measures for the assessment of anxiety are able to identify which children will be highly anxious during induction of anesthesia. Examples of such measures are the Screen for Child Anxiety Related Disorders (SCARED) (Birmaher et al. 1997) and Multidimensional Anxiety Scale for Children (MASC) (March et al. 1997). However, a disadvantage of most narrow-band measures for the assessment of anxiety may be that these are focused on disorders (i.e. on DSM criteria). As an alternative, one option could be to specifically assess hospital-related anxiety, for example using the pediatric index of emotional distress (PI-ED) (O’Connor et al. 2016), which is modeled on the hospital anxiety and depression scale (HADs) for adults (Zigmond and Snaith 1983). Future studies are needed to investigate whether narrow-band and hospital-related anxiety instruments would be able to detect anxiety during induction of anesthesia in children.

We hypothesized that state anxiety at hospital arrival would be a stronger predictor of anxiety during induction than internalizing behavior during the past 6 months, because of a closer temporal association. Our results confirmed this hypothesis, as higher parent- and observer-reported state anxiety at hospital arrival were significantly associated with anxiety during induction of anesthesia. This finding is in line with previous studies that found anxious behavior in the direct preoperative period was related to anxiety during induction of anesthesia (Chorney and Kain 2009; Kain et al. 2007). However, at that point, shortly before entering the operating room, anxiety may already have increased significantly. Moreover, not much time is left for professionals or parents to take action at this stage. Consequently, it is important to assess the risk of experiencing intense levels of anxiety during induction early on the day of surgery, i.e. already at hospital arrival. This may allow healthcare professionals to intervene before anxiety increases.

Different non-pharmacological interventions that can be applied on the day of surgery have been shown to reduce anxiety during anesthesia induction in pediatric patients. For example, Brewer et al. (2006) found that the psychological preparation of children (aged 5 to 11 years) for elective surgery by a child life specialist was associated with decreased preoperative anxiety. That preparation included exploring and rehearsing with anesthetic equipment, such as the anesthesia mask and pulse oximeter (Brewer et al. 2006). Another well-known intervention during induction of anesthesia is to provide distraction. By directing attention away from the procedure and redirecting it to something fun and engaging, less attention is available for the perception of anxiety (Kleiber and McCarthy 2006; Lambert 1996). Examples of distraction techniques used during induction of anesthesia are playing hand-held videogames, watching cartoons or videos, distraction by parents or nurses, playing with toys, and listening to music (Kain et al. 2004a, b; Koller and Goldman 2012; Lee et al. 2012; Patel et al. 2006). An innovative intervention to improve induction of anesthesia in children is providing a virtual reality tour of the operating room environment and the anesthetic procedures, prior to surgery (Eijlers et al. 2019a, b; Ryu et al. 2017, 2018). Using virtual reality to prepare children for what they will see and hear, preoperative anxiety and its postoperative consequences could be reduced. However, according to a recent meta-analysis of virtual reality interventions for pediatric patients, more research is needed to ascertain the effectiveness of virtual reality preparation for surgery (Eijlers et al. 2019a, b).

In the current study, we used the STAIC for the differentiation between children who will and will not be highly anxious during anesthesia induction, since we found that this assessment tool was the strongest predictor at hospital arrival for anxiety during induction. ROC analyses indicated a STAIC score of 47.50 as the optimal cut-off value to distinguish high-risk patients (associated with a mYPAS score during induction of 67.79) from other patients. In previous studies, mYPAS scores of 30 and 35 have often been used as cut-off values (based on a self-reported STAIC reference score of 37) (Kain et al. 1997). For example, recently, Malik et al. (2018), using 30 as the mYPAS cut-off score, found that 48% of the 7- to 12-year-old children in their study were anxious shortly before entering the operating room, 72% at parental separation, and 95% during induction. These results indicate that anxiety increases for a significant number of children between hospital arrival and induction of anesthesia. While this is true, this approach ignores the fact that there are considerable variations in the levels of anxiety experienced by children within that 95%. Even though the majority of children have mYPAS scores around 30 or 35, we argue that it is important to focus on those children who experience intense levels of anxiety, as they are considered to be at the highest risk of maladaptive postoperative consequences, such as pain, emergence delirium, and sleeping disturbances (Chorney and Kain 2009; Kain et al. 2004a, b).

Implementing the parent-reported version of STAIC is relatively easy, given it only takes a few minutes to complete the questionnaire and no hospital staff is needed for the assessment of anxiety. Moreover, involving parents in the health care of their children is in line with family-centered care (Kuhlthau et al. 2011). The results of this study indicate that completing the STAIC at hospital arrival allows parents, anesthesiologists, and other healthcare professionals, such as (daycare) nurses or child life specialists, to differentiate between children who will experience intense levels of anxiety during induction and children who will not. Distinguishing between the two categories at this early stage makes more effective targeted interventions possible. This study can also contribute to future studies investigating interventions to reduce preoperative anxiety by indicating how to assess and therefore target those children at highest risk.

### Clinical Implications

The results of this study are of clinical importance, as completing the STAIC at hospital arrival may help nurses, anesthesiologists, and other clinical personnel identifying highly anxious children. It is well known that preoperative anxiety increases during the day of surgery and peaks during induction of anesthesia (Chorney and Kain 2009; Davidson et al. 2006; Kain et al. 2007, 1996). Therefore, being able to predict at an early stage, already at hospital arrival, which children will experience intense levels of anxiety during induction of anesthesia is extremely valuable. Using the STAIC at hospital arrival may make it possible to prevent anxiety from escalating by providing the psychological interventions as described above, such as introducing the child to the anesthetic procedures by rehearsing with anesthetic equipment (Brewer et al. 2006). We advise using the STAIC as a decision tool to determine whether to apply certain interventions depending upon the specific needs of the child. This may improve compliance during induction and decrease the possible negative short-term and long-term impact of anxiety during induction of anesthesia.

Most children experience some levels of anxiety during induction of anesthesia and intense levels of anxiety in particular are associated with maladaptive consequences (Kain et al. 2004a, b; Kain et al. 1996; Power et al. 2012). Therefore, as discussed above, we contend that it is important for clinical practice to apply a cut-off score for the STAIC that corresponds with levels of intense anxiety as indicated by the mYPAS. This may help healthcare professionals to pay extra attention to high-risk children. Furthermore, targeting interventions to subgroups of patients, i.e. to patients with high levels of anxiety at hospital arrival, may improve the efficacy of psychological interventions to reduce anxiety during induction of anesthesia.

### Strengths and Limitations

The strengths of this study include the large sample size, the use of well-validated and easily implemented assessment tools, and its attention to predictors of anxiety during anesthetic induction.

Certain limitations also need to be addressed. First, the children did not complete the STAIC themselves. We used parent reports instead, which could have been influenced by parental anxiety. However, it should be pointed out that reports filled out by children themselves also have validity problems, especially where young children are concerned, because a certain level of cognitive development is needed, particularly in stressful situations (Schisler et al. 1998). Second, the current study only included children undergoing elective daycare surgery. Therefore, the applicability of the current results to other patient groups has yet to be vindicated.

## Conclusion

Most children experience anxiety on the day of surgery, but with a wide range of intensity. At hospital arrival, the CBCL was not a significant predictor of anxiety during induction of anesthesia but the STAIC and the mYPAS were. The STAIC (state form) was considered the strongest predictor and could be used to predict intense levels of anxiety during anesthetic induction. Applying this easy-to-use tool at hospital arrival allows children at high risk to be identified, resulting in the potential for focused interventions by healthcare professionals before induction of anesthesia. By reducing the anxiety of the child a smoother induction may well be possible. However, more research is needed, especially with respect to other patient groups.
